# Air Pollution: A Neglected Risk Factor for Dementia in Latin America and the Caribbean

**DOI:** 10.3389/fneur.2021.684524

**Published:** 2021-07-22

**Authors:** Nathália Villa dos Santos, Victor Yuji Yariwake, Karina do Valle Marques, Mariana Matera Veras, Laís Fajersztajn

**Affiliations:** ^1^Laboratório de Poluição Ambiental, Departamento de Patologia, Faculdade de Medicina, Universidade de São Paulo, São Paulo, Brazil; ^2^Departamento de Saude Ambiental, Faculdade de Saude Publica, Universidade de São Paulo, São Paulo, Brazil; ^3^Departamento de Cirurgia, Universidade Federal de Uberlandia, Uberlândia, Brazil

**Keywords:** air pollution, particulate matter, dementia, Alzheimer, Latin America, low-and-middle income countries, developing countries

## Abstract

The risk of dementia and Alzheimer's disease in Latin America and the Caribbean (LAC) rises with increasing age and polluted air. Currently, at least 172 million people breathe unhealthy levels of air pollution in LAC countries. Several cohort studies have indicated that air pollution increases the risk of developing dementia and neurodegenerative diseases, but the mechanisms underlying the association are still not clear. Air pollution causes and aggravates five established risk factors for dementia (obesity, hypertension, stroke, diabetes mellitus, and heart diseases) and is linked to three other risk factors (physical inactivity, cognitive inactivity, and depression). Some of these risk factors could be mediating the association between air pollution and dementia. Reducing the risks for dementia is crucial and urgently needed in LAC countries. There is room for improving air quality in many urban areas in the LAC region and other low- and middle-income countries (LMICs), a routealready explored by many urban areas in developing regions. Moreover, reducing air pollution has proved to improve health outcomes before. In this article, we propose that despite the ongoing and valid scientific discussion, if air pollution can or cannot directly affect the brain and cause or aggravate dementia, we are ready to consider air pollution as a potentially modifiable risk factor for dementia in LAC and possibly in other LMICs. We suggest that controlling and reducing current air pollution levels in LAC and other LMIC regions now could strongly contribute.

## Introduction

Despite the ongoing and valid scientific discussion about whether air pollution can or cannot directly affect the brain, and cause or aggravate dementia, in this article, we propose that we are ready to consider air pollution as a modifiable risk factor for dementia in Latin America and the Caribbean (LAC), and in other low- and middle-income countries (LMICs). We argue that controlling and lowering current air pollution levels in these regions could strongly contribute to reducing the high burden of dementia cases that has been projected for these countries. We base our argument on the following findings: 1) air pollution causes and/or aggravates at least five of the established risk factors for dementia (i.e., obesity, hypertension, stroke, diabetes mellitus, and heart diseases), 2) reducing air pollution has been proven to lower some of these risks, and 3) there is room for improving air quality in many urban LAC areas and other LMICs, a path already explored in many urban developing regions. First, we will introduce the topic and our hypothesis. In the following subsections, we will discuss how air pollution causes obesity, hypertension, stroke, diabetes mellitus, and heart diseases, and how these conditions can lead to dementia. We will also discuss the potential relations between air pollution and three other risk factors for dementia, for which evidence is less robust (physical inactivity, cognitive inactivity, and depression). Finally, we will discuss the evidence supporting the idea that air pollution can directly cause dementia. In the discussion, we will address why controlling air pollution should be used as a risk reduction strategy to tackle the burden of dementia in the LAC region and LMICs.

## Air Pollution as a Modifiable Risk Factor for Dementia in Latin America and the Caribbean

### Dementia

Dementia is an expensive disease with no cure to date, and its estimated global cost has already exceeded 1 trillion dollars. This is an extraordinarily high number for LMICs, a region that will concentrate 68% of the dementia cases by 2050 (1).

The burden of dementia in LAC countries is high and will continue to rise. By 2050, the region will have more than 17 million dementia cases, a 4-fold increase in 35 years (1). The average world increase for the same period will be three times lower (1). Other reports have described how rapid demographic changes, a higher prevalence of risk factors for dementia in the population, and other specific issues have made dementia prominent in the LAC region (2–4). Risk reduction is a crucial public health response to dementia (5), one that is urgently needed in the LAC countries. Our message is that air pollution could figure among the risk reduction strategies in LAC.

### Air Pollution

Air pollution is a complex mixture of particles and gases in the air. Its composition varies depending on the emission source and prevailing weather conditions (6). Fine particulate matter [PM with an aerodynamic diameter of ≤ 2.5 μm (PM2.5)] is frequently presented as an indicator of exposure to air pollution in health studies (7, 8), including studies on the effects of air pollution on dementia (9–14). Rather than a single pollutant, PM2.5 reflects different sources of air pollution in the atmosphere. Because of the tiny particle size, PM2.5 can penetrate the lung barrier and enter the blood system. No matter its composition, PM is considered carcinogenic (15). The most frequent gas used to indicate exposure to air pollution in studies investigating the effects of air pollution on dementia is nitrogen dioxide (NO_2_), a marker of vehicle exhaust (11–14, 16). Some specific heavy metals, such as lead and cadmium, can trigger neurodegenerative processes and dementia (17). Widely used in industrial and agricultural activities, heavy metals are present in the soil and water and can affect the air, too. Once in the air, they become a major occupational health concern and a threat to the surrounding population. It is crucial to identify the sources of heavy metal air pollution and prevent hazard exposures.

Air pollution is ubiquitous and unevenly distributed (18–20), but it can be reduced. From a global perspective, pollution levels are higher in LMICs (20), the ones that will concentrate approximately 80% of dementia cases in the coming years (1). This makes tackling air pollution a relevant strategy to promote equity in brain health. The unequal distribution of air pollution is also true at smaller scales. Within the same city, those living closer to high traffic roads breathe worse air quality than those living further away. The same goes for those of lower socioeconomic status (21–23).

In 2015, air pollution caused 7.6% of the total global deaths and 4.2% of the total global disability-adjusted life-years (DALYs), a measure of loss in quality of life (7). This estimation was based on the risk for selective cardiovascular (CVD) and respiratory diseases, with no cognitive impairment burden included. It is reasonable to assume that a great part of this burden could be reduced just by lowering levels of air pollution. Indeed, some studies have used natural experiments to show that not only does bad air quality impair health, but also good air quality improves it (24–27). In other words, reducing air pollution can reduce global premature mortality and enhance the quality of life.

### Air Pollution in Latin America and the Caribbean

Air pollution in LAC countries does not receive much attention on the global stage (19, 20). This is probably because, when compared with Africa, Asia, and the Middle East, air pollution in this region is not very high (18). However, between 1990 and 2013, particulate pollution increased in parts of the LAC territory (18). If one considers the current knowledge on pollution control and the performance of high-income countries, the decreases in the air pollution levels in LAC countries have been modest. From a public health perspective, the slight reduction in pollution in some regions of LAC has been offset by population growth (7). Although levels of fine particulate matter were decreasing since 2010 in some of the larger LAC cities, such as São Paulo and Bogotá, the current trends point towards an increase (28). At least 172 million people breathe unhealthy levels of air pollution in the LAC region, according to a recent estimation including 100,000 urban residents (29). The total number of LAC residents breathing bad air is higher if we include the rural residents, and remember that the LAC population is nearly 65 times larger than the population used in this estimate. Some rural LAC areas are highly polluted (18) because of wildfires (intentional or not) (30, 31), preharvest sugar cane burning (32, 33), and the more frequent use of wood for cooking and heating (34). At least 172 million LAC residents could reduce their risk for several diseases if air quality improved.

### Hypothesis

Reducing the risk of dementia in LAC countries is urgent. The World Health Organization (WHO) strongly recommends that countries take national actions to lower well-established risk factors for dementia at the individual and population levels (5). These risk factors include obesity, hypertension, stroke, diabetes mellitus, heart disease, physical inactivity, poor diet, alcohol abuse, tobacco use, cognitive inactivity, depression, and isolation (5, 35–37). At least five of these risks can be caused or aggravated by breathing bad air quality. This means that controlling air pollution through policies and regulations could help reduce the burden of dementia. This is particularly relevant in LAC countries, where cases will increase, and pollution control is not typically stringent. Large urban areas of the most populated LAC territories, such as São Paulo and Mexico City, present poor air quality far above the health safety levels recommended by the WHO. Both these metropolitan regions rank among the global cities with the highest traffic intensity indexes. Reducing traffic intensity is an example of a pollution control policy that has already been successfully adopted in other large urban areas in Europe, for example.

Recently, mechanistic studies have suggested that air pollution particles can cross the olfactory barrier and reach directly the brain (38). Once in the brain, these particles could trigger neuroinflammation (39, 40) and exaggerated protein misfolding, leading to dementia (9, 40–42). This evidence is still in its early stages and is not yet robust. However, a growing number of epidemiological studies in Europe (11, 13, 16, 43), North America (9, 12, 14, 44–46), and Asia (10) are showing that living in highly polluted areas increases the risk of dementia. Some studies suggest that the link is driven by cardiovascular alterations caused by air pollution (13, 14), but the direct effect pathway was not ruled out. The hypothesis that the association between air pollution and dementia is mediated by cardiovascular disease and metabolic syndrome is reasonable since air pollution is known to cause and aggravate cardiovascular conditions. Nevertheless, the scientific community does not seem to be ready to affirm that air pollution affects the brain and causes dementia (47). Studies in the field are susceptible to a wide range of biases, from the selection of dementia cases to the characterization of long-term exposure to air pollution for each participant. This might explain why air pollution has not yet been included in the list of established and potentially modifiable risk factors for dementia (35–37). We suggest that it is important to change this perspective and consider air pollution a risk factor for dementia, to reduce the burden of the disease in LAC countries. Furthermore, this should be done posthaste. We are not alone. In August 2020, for the first time, a report on dementia prevention listed air pollution as a modifiable risk for the condition (48).

## Indirect Associations: Chronic Diseases Linked to Dementia and Air Pollution

Air pollution causes and/or aggravates obesity, hypertension, stroke, diabetes mellitus, and heart diseases. These are all established risk factors for dementia (35–37). Air pollution has also been linked to three other established risk factors for dementia (i.e., physical inactivity, cognitive inactivity, and depression), but the evidence is weaker. In this section, we will discuss these links.

### Obesity, Diabetes Mellitus, and Physical Inactivity

Approximately 302 million adults living in the LAC region are overweight, and more than 100 million are obese (49). By the year 2030, overweight and obesity are expected to affect 50% of men and 60% of women in Latin America (50). The countries with the highest prevalence of obesity are El Salvador and Paraguay for females, and Uruguay and Chile for males (51).

Overweight and obesity can be reversed and prevented. Air pollution is linked to metabolic disorders (52). For this reason, safe air quality is among the actions that may reduce the burden of overweight and obesity, alongside healthy diets and physical exercise. Various mechanisms underlie the link between air pollution and increased body weight. Air pollution can cause metabolic dysfunction by increasing oxidative stress in adipose tissue (53) and inflammation. Besides this, liver cells can accumulate more lipids, and skeletal muscles may use less glucose. Obesity is a significant health challenge, as it substantially increases the risk for diseases, such as type 2 diabetes mellitus, fatty liver disease, stroke, and dementia (54). More recently, obesity has been identified as a risk factor for Alzheimer's disease due to hyperglycemia (55). Air pollution can indirectly affect body weight, thus, increasing the risk of other chronic diseases.

Air pollution also prevents people from engaging in regular physical activity, resulting in sedentary behavior (56, 57). Significant levels of air pollution have been negatively associated with physical activity due to decreased lung function, high blood pressure, and other cardiovascular and respiratory symptoms that impair exercise capacity and performance (56, 57).

### Cardiovascular and Cerebrovascular Diseases

Epidemiological and observational studies have deepened the knowledge we have about air pollution and its effects on human health (58). Cardiovascular diseases and high blood pressure have been associated with short and long term exposure to air pollution, generated by inflammation, oxidative stress, and arterial remodeling (58, 59).

There are two main types of exposure to air pollution. The first is acute exposure, defined as short-term exposure or a slow but brief increase in pollutant concentrations that may vary in a single day or over several days. Chronic or long-term exposure is exposure to air pollutants over several months or years. Acute exposure to air pollution can result in a similar acute event, such as a thrombosis-induced stroke (60), a rapid rise in blood pressure, or cardiac arrhythmia. Chronic exposure to air pollution can have a direct effect on the brain, triggering and/or furthering neurodegeneration (61) or silent ischemic injuries (62).

Short-term exposure to air pollution can induce vasoconstriction, rupture of atheromatous plaques through inflammatory processes, and cause oxidative stress and thrombin activation. Regarding PM2.5 concentrations, these may cause reduced blood flow in the brain, and ozone can induce cardioembolic strokes, affect heart rate variability, and stimulate atrial fibrillation. On the other hand, chronic exposure can manifest clinically as recurrent strokes provoked by the genesis and progression of atheromas and myocardial infarction (63).

Exposure to air pollution can trigger changes in the cardiovascular system, and these effects can develop acutely, with increased atherosclerosis accelerating the response to chronic disease. Air pollution has also been related to lowering the density of high-density lipoproteins (HDL) due to oxidative stress and inflammation. These processes can cause changes in the structure and function of HDL, resulting in pro-atherogenic or dysfunctional HDL (64). Furthermore, air pollution has been correlated with an increased risk for ventricular arrhythmias and increased ventricular electrical instability, highlighting the potential link between air pollution and sudden cardiac deaths (65).

High concentrations of air pollutants may be correlated with the risk for angina and acute myocardial infarction. Short-term exposure to PM2.5 at levels of 10 mg/m^3^ or higher contributes to acute coronary syndromes, especially in patients with preexisting heart disease. The effects are more severe after long-term exposure to traffic (66, 67). Recent studies have suggested that even modest increases in airborne pollutants can trigger an increase in blood pressure in a few hours (68) and affect heart rate variability, vascular tone, and blood coagulation, as well as promote atherosclerosis (69). Even short-term exposure to air pollution has been associated with increased cardiovascular issues and deaths from myocardial ischemia, arrhythmia, and heart failure (59, 70, 71).

Hypertension and heart disease are the main diseases that affect the population worldwide (72). Several studies have indicated that air pollution is a risk factor for hypertension, heart disease (73, 74), and stroke (72, 73).

Research has produced some evidence regarding the association between hypertension, changes in blood pressure, and exposure to PM2.5 and PM10 (particles with an aerodynamic diameter of_or greater than 10 μm). Results indicated that long-term exposure to a 10-μg/m^3^ increase in PM10 and PM2.5 is significantly associated with higher systolic and diastolic blood pressure, in addition to a greater risk of hypertension (74). The hypertensive effects of air pollution were more pronounced among men, smokers, drinkers, individuals with a high-fat diet, and those with a high level of physical activity (75).

Elderly patients demonstrate a more apparent association between high blood pressure and cognitive decline when the former condition first arises in middle age. Hypertension can promote changes in the structure and function of the brain through remodeling cerebral vessels. This can lead to disruptions in cerebral self-regulation, reductions in cerebral perfusion, and limitations in the brain's ability to clear potentially harmful proteins, such as β-amyloid (76). Moreover, hypertension disrupts the structure and function of brain blood vessels and can lead to ischemic damage in the white matter that is critical for cognitive function, thus, favoring the development of neurodegenerative diseases (77).

In the case of cerebrovascular diseases, exposure to air pollution particles has also been associated with neuroinflammation (78). The first evidence of neuroinflammation caused by the inhalation of pollution was published in a study involving dogs that had been exposed to significant concentrations of ozone, PM, and other pollutants. Researchers detected inflammation in the brain of the animals with endothelial damage (79). Other *in vivo* studies have shown that PM can stimulate inflammatory and oxidative responses in the CNS (80, 81). Mice exposed to diesel inhalation showed acceleration in the development of Alzheimer's disease characteristics (82), and activated microglial and inflammatory responses. These animal studies demonstrated signs of cellular damage associated with air pollution (83, 84).

## Air Pollution, Depression, and Cognitive Inactivity

Studies have shown that exposure to air pollution may be associated with more frequent incidence of depression, anxiety, and cognitive inactivity, especially in people who suffer from concomitant chronic diseases (85). Another study has shown that increases in air pollution may lead to the onset of depressive symptoms among the elderly (41). The pathogenesis may involve oxidative stress and generalized inflammation induced by PM. The pathomechanisms have been linked to vascular lesions and neurodegenerative disorders (86). As a result, these processes may cause or exacerbate the symptoms of depression (87).

## Toxicity Mechanisms of Air Pollution

Exposure to contaminated external air is considered an environmental risk factor that promotes brain aging; in contrast, the effects of air pollution on the central nervous system (CNS) are not broadly recognized. Air pollution can cause various neurological disorders as a result of nervous system inflammation and oxidative stress. Damage to neuronal cells caused by fine dust, especially in fetuses and infants, can cause permanent brain damage or lead to neurological disease in adulthood. Epidemiological studies have reported that the risk of developing dementia and Alzheimer's disease is increased by exposure to fine particulates (60).

The role of air pollution in causing metabolic dysfunction is already known (53). The mechanisms by which air pollutants affect the body systems are intimately related to the type and size of inhaled pollutants. The size, load, chemical composition, and propensity to form aggregates determine a pollutant's ability to cross the lung and the blood barrier. The large-diameter PM (PM10) cannot be transported directly into the blood circulation and require neural or proinflammatory responses to mediate extrapulmonary actions, whereas the ultrafine or soluble PM that constitutes smaller particles can enter the bloodstream directly.

**Figure 1:**
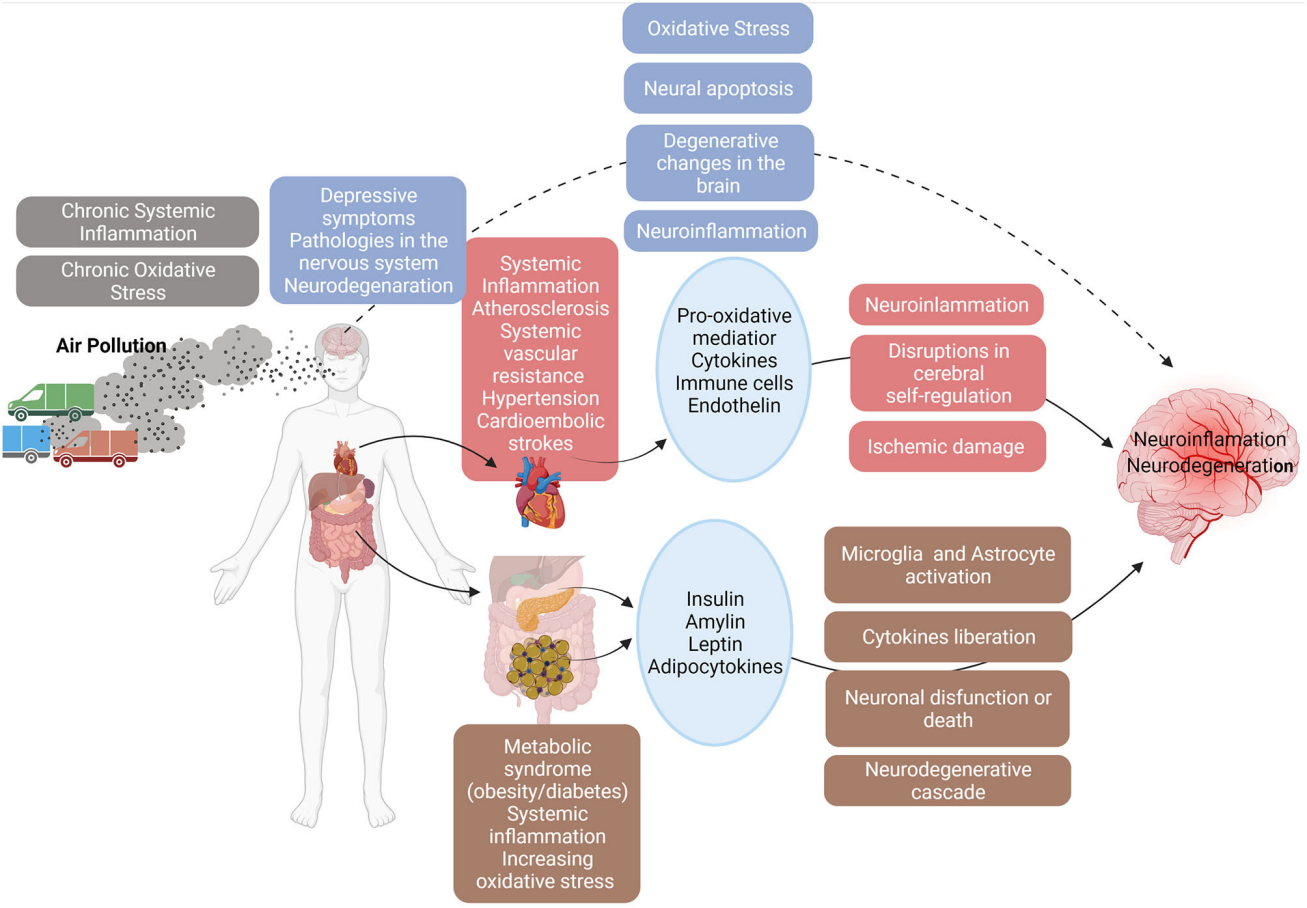
The toxicity mechanisms: air pollution acts directly in brain and indirectly in cardiovascular diseases and metabolic syndromes causing neuroinflammation and neurodegeneration. Created with BioRender.com.

In Figure 1, we show the plausible mechanisms of how air pollution affects the human health leading to neurologic impairments highlighting the increased oxidative stress and the pronounced inflammatory responses associated with cardiovascular diseases and metabolic syndromes.

The toxic effects of pollution on cardiovascular diseases are also known (58, 59). The first response caused by inhalation of air pollutants is characterized by the release of pro-oxidative/inflammatory mediators (cytokines, activated immune cells, or platelets) and/or vasoactive mediators (endothelin) in the systemic circulation. Particle inhalation can trigger systemic inflammation, atherosclerosis, and endothelial dysfunction, resulting in increased systemic vascular resistance and hypertension. It is worth noting that the duration of exposure, the levels of co-pollutants, and patient sensitivity are factors that determine subsequent responses (70, 71).

In the second path of action, air pollutants accumulate in the respiratory tree. This can directly stimulate the lung's nervous reflexes, leading to an autonomic imbalance that subsequently favors vasoconstriction (70, 71). In the third pathway, metallic particles and ultrafine PM are inhaled. These can pass through the alveolar and capillary membrane, reach the circulatory system, and cause damage to vasomotor regulation (58, 70, 71, 88).

The direct or indirect effects of air pollution can be associated with stroke and dementia risks. Direct damage can be caused by neuroinflammation and neurodegeneration leading to neurodegenerative diseases (38). Magnetite findings in the magnetic resonance images of human brains exposed to air pollution explain the direct relationship between PM air pollution and AD (89). The indirect mechanism of air pollution in the cerebrovascular system causes stroke, vascular dementia, or other types of dementia. This may be mediated by certain effects on the cardio-neuro-vascular system, such as atherosclerosis, plaque formation and rupture, endothelial dysfunction, cardiac arrhythmia, hypertension, and activated thrombin (90, 91).

By itself, the obesity–diabetes–dementia connection occurs through inflammation and oxidative stress in the brain, caused by systemic inflammation. It is characterized by increased levels of systemic inflammatory mediators (cytokines) that cross the blood–brain barrier (BBB) and metabolic mediators released by adipose tissue and the pancreas (leptin, insulin, and amylin) that play additional important roles in mediating central nervous system inflammation and neuronal regulation (92), and leading to activation of microglia and astrocytes, inducing the release of cytokines and oxidants. This inflammatory microenvironment causes neuronal dysfunction or death and creates a neurodegenerative cascade.

In obesity and diabetes, there is evidence that exposure to PM 2.5 alters endothelial function, increasing serum levels of tumor necrosis factor alpha (TNF-a), as well as higher levels of interleukin-6 (IL-6), resistin, and leptin (93). Furthermore, Gasparotto et al. (94) investigated oxidative damage and inflammation in different brain regions of obese rats exposed to coal ash, and the results showed that obese rats that inhaled coal ash were more affected by oxidative damage with subsequent inflammation in the hippocampus. This type of damage can lead to chronic neurodegeneration (94).

There are many mechanisms through which air pollutants may cause pathologies in the nervous system, such as nervous system inflammation, oxidative stress, microglial cell activation, protein condensation, and cerebral vascular barrier disorders, but how these mechanisms occur is still unclear (60). The main components of air pollution, PM2.5, as well as the compounds absorbed on their surfaces arrest cell cycles and cause neural apoptosis. These effects, together with the oxidative stress and gene expression alteration induced by these particles, may lead to degenerative changes in the brain (95). Substances found in smog may also cause systemic inflammation with an increased number of active immune cells producing proinflammatory cytokines that pass through the BBB via active transport. As a result, the migration of monocytes to the central nervous system is intensified, aggravating the neuroinflammation process (96).

## Direct Association: Air Pollution and Dementia

Higher prevalence of neurodegenerative diseases will certainly come along with population aging. Taking dementia as a reference, the higher increase will occur in LMICs (97).

Air pollution represents an important source of premature mortality and morbidity globally (98) but unevenly distributed across the Globe, since household and outdoor air pollution concentrations are higher in developing countries (20). Adverse effects of air pollution on human health have been mostly attributed to cardiorespiratory events. However, recent studies provide evidence that other health conditions may be influenced by exposures to airborne contaminants, including central neurodegenerative diseases such as Alzheimer's disease, Parkinson's disease, and mood disorders (99–101).

A series of studies conducted in Mexico demonstrated the usefulness of autopsies in exploring the links between air pollution and cerebral damage (38, 81, 87–89, 102). Thus, a study combining precise measures of exposure, collected cerebral tissues, and autopsy materials could provide important information about the role of air pollution in the development of neurodegenerative diseases.

Other researchers investigated the distribution of the alpha-emitting radon progeny 210-Polonium (^210^Po) in the olfactory epithelium, olfactory bulb, frontal lobe, and lung tissues of cadavers in São Paulo, Brazil (103). The findings showed that the olfactory bulbs presented higher concentrations of ^210^Po in comparison with other tissues. The higher concentrations of ^210^Po in olfactory bulbs suggest that this is a major path entryway for transporting radon progeny from nasal tissues to the central nervous system.

In children, respiratory inflammation is recurrent, especially in the nasal epithelium (99). This leads to changes in the nasal mucous membranes, causing the breakdown of the nasal epithelial barrier and facilitates the passage of xenobiotics into the systemic circulation and the brain. Such damage causes neuroinflammation and the breakdown of the BBB and can affect the olfactory bulb, prefrontal cortex, and brain stem. In patients with apolipoprotein E4, it can significantly accelerate olfactory decline, attention, and short-term memory contributing to neurodegeneration and the progress of Alzheimer's disease (80, 104, 105).

## Discussion

The burden of dementia in LAC is high and will increase (1). Reducing dementia risks is crucial and urgently needed in this region. Reducing the levels of air pollution has been shown to improve health outcomes (24–26, 106). Several parts of the LAC region experience bad air quality, and some areas will see an increasing trend in air pollution levels in the coming years (18, 28). At least 172 million LAC inhabitants breathe unhealthy levels of air pollution (29), but there is room to improve their air quality. A better air quality in the region could reduce their risk of developing dementia.

Air pollution control and regulations in LAC countries are not robust (107) and could be improved. Official ground-level information on pollutants is rare (107), and only eight countries have set National Air Quality Standards for fine particulates (108), all above the level recommended by the World Health Organization (109). Three countries in the LAC region were studied in a systematic review on interventions to improve air quality (106), but the reported policies have had little to no success in improving pollution levels. In Ecuador, government action during highly polluted periods effectively reduced the levels of particulate pollution by 20% (110). Although this is a positive policy that will prevent peaks in air pollution, it provides little to no aid in managing the hazards of long-term exposure to polluted air. In Chile, reducing the number of buses alone was not effective in reducing pollution (111). The reported reforms in the public transportation systems in Mexico City and Santiago resulted in a large number of cars and higher levels of pollution (112). Despite the scarcity of documented reports in the literature, the region has had successful experiences controlling air pollution. A proof is the reduction in fine particulate pollution after 2010 in São Paulo and Bogotá (18). At least in São Paulo, the better air quality is mostly attributed to a national pollution control program in Brazil that set progressively more stringent standards to reduce vehicular emission of new models and the large scale of ethanol and biodiesel enforced by law (113). The targets and deadlines of the Brazilian pollution control program were postponed in different occasions along the years. It is important to notice that the improvement in air quality was achieved for some, but not all, pollutants in the air. The control of secondary pollutants (pollutants formed in the atmosphere, not directly emitted from a source, such as fine particulates) remain a challenge, and despite the improvement in air quality, pollution levels in São Paulo do not yet conform to the WHO's safety recommendations (113). Pollution control and management in São Paulo and in LAC need to be more aggressive to achieve the air pollution safety levels recommended by the WHO. A discussion on possible interventions to improve air quality and health outcomes can be found elsewhere (106).

As explained in detail in previous sections of this manuscript, air pollution causes and aggravates five established risk factors for dementia (obesity, hypertension, stroke, diabetes mellitus, and heart diseases) and has been linked to three others physical inactivity, cognitive inactivity, and depression (35–37). CVD and diabetes mellitus (DM) are the leading causes of mortality and morbidity in the LAC region, resulting in almost 1 million deaths. In 2016, 18.4 million DALYs were lost to cardiovascular disease (114, 115). For diabetics, there were 195,000 deaths, and 8.5 million DALYs were lost in 2016 (114–117). In 2019, these numbers improved by 1% for both diseases (114). The prevalence of these risk factors is higher in LAC countries when compared with the world and other LMICs. The population attributable fractions (PAF) for hypertension is 9% in the LAC region vs. 2% around the world, 6% in China, and 4% in India. The obesity numbers show a similar situation: 8% in the LAC territory vs. 1% around the world, 6% in China, and 3% in India. For diabetes, the LAC PAF is 3% vs. 1% around the world, and 2% in China and India (118). Fifty-six percent of the dementia cases in LAC countries could be avoided if individual risk factors were eliminated. That proportion is higher than in China (49%) and India (41%) (118).

Reducing air pollution could attenuate the prevalence of these established risk factors in the population and, therefore, lower the high burden of dementia in the region. Indeed, two recent cohort studies in Europe (13) and North America (14) have suggested that cardiovascular diseases mediate the association between air pollution and dementia. No similar studies regarding LAC countries were found. However, there is no reason to speculate that cardiovascular disease triggered by air pollution would not culminate in dementia in the LAC population.

As we mentioned in the *Introduction* section, it is plausible to think that air pollution may directly cause dementia. The body of evidence on air pollution and dementia is growing rapidly, with many relevant studies published recently (13–16). Further studies will collaborate to elucidate if air pollution can enter the brain and cause dementia. Nevertheless, whether the agency is direct or indirect, it is clear that air pollution has been associated with dementia, and it is reasonable to expect that reductions in air pollution would help decrease the burden of this disorder.

Given the urgent need to lower the burden of dementia in LAC countries, we recommend implementing strategies to reduce air pollution levels and related risks. We base our argument on the findings that (1) air pollution causes and/or aggravates at least five of the established risk factors of dementia (obesity, hypertension, stroke, diabetes mellitus, and heart diseases), (2) reducing air pollution has been shown to reduce some of these risks, and (3) there is room for improving the air quality in many urban LAC areas and other LMICs. These improvements are already being explored by several urban centers in developing regions with proven health benefits for their population. Although we will just be possible to estimate the real impact of reducing the levels of air pollution in LAC in the burden of dementia in the future, there is no reported side effect for breathing better air quality.

## Data Availability Statement

The original contributions presented in the study are included in the article/supplementary material, further inquiries can be directed to the corresponding author/s.

## Author Contributions

LF designed the study, reviewed the literature, and wrote the manuscript. NVS and MMV reviewed he literature and wrote the manuscript. VYY interpreted and synthetize the data and revised the manuscript. KVM updated the literature review and revised the manuscript. All authors approved the final version of the manuscript and agreed to be accountable for all aspects of the work.

## Conflict of Interest

The authors declare that the research was conducted in the absence of any commercial or financial relationships that could be construed as a potential conflict of interest.

## References

[1] International A-AsD. World Alzheimer Report 2015. The Global Impact of Dementia: an Analysis of Prevalence, Incidence. Costs and Trends. London: Alzheimer's Disease International (ADI). (2015).

[2] Custodio N Wheelock A Thumala D Slachevsky A. Dementia in Latin America: epidemiological evidence and implications for public policy. Front Aging Neurosci. (2017) 9:221. 10.3389/fnagi.2017.00221 28751861 PMC5508025

[3] Ibanez A Parra MA Butlerfor C Latin A the Caribbean Consortium on D. The latin america and the caribbean consortium on dementia (LAC-CD): from networking to research to implementation science. J Alzheimers Dis. (2021) 82:S379–S394. 10.3233/JAD-201384 33492297 PMC8293660

[4] Parra MA Baez S Allegri R Nitrini R Lopera F Slachevsky A. Dementia in latin America: assessing the present and envisioning the future. Neurology. (2018) 90:222–31. 10.1212/WNL.0000000000004897 29305437 PMC5791795

[5] Organization WH. Global Action Plan on the Public Health Response to Dementia 2017–2025. Geneva: World Health Organization. (2017).

[6] Adams DR Ajmani GS Pun VC Wroblewski KE Kern DW Schumm LP. Nitrogen dioxide pollution exposure is associated with olfactory dysfunction in older U.S. adults. Int Forum Allergy Rhinol. (2016) 6:1245–52. 10.1002/alr.21829 27620703 PMC5554588

[7] Cohen AJ Brauer M Burnett R Anderson HR Frostad J Estep K. Estimates and 25-year trends of the global burden of disease attributable to ambient air pollution: an analysis of data from the Global Burden of Diseases Study (2015). Lancet. (2017) 389:1907–18. 10.1016/S0140-6736(17)30505-6 28408086 PMC5439030

[8] Forouzanfar MH Afshin A Alexander LT Anderson HR Bhutta ZA Biryukov S. Global, regional, and national comparative risk assessment of 79 behavioural, environmental and occupational, and metabolic risks or clusters of risks, 1990–2015: a systematic analysis for the Global Burden of Disease Study 2015. Lancet. (2016) 388:1659–724. 10.1016/S0140-6736(16)31679-8 27733284 PMC5388856

[9] Cacciottolo M Wang X Driscoll I Woodward N Saffari A Reyes J. Particulate air pollutants, APOE alleles and their contributions to cognitive impairment in older women and to amyloidogenesis in experimental models. Transl Psychiatry. (2017) 7:e1022. 10.1038/tp.2016.280 28140404 PMC5299391

[10] Jung CR Lin YT Hwang BF. Ozone, particulate matter, and newly diagnosed Alzheimer's disease: a population-based cohort study in Taiwan. J Alzheimers Dis. (2015) 44:573–84. 10.3233/JAD-140855 25310992

[11] Carey IM Anderson HR Atkinson RW Beevers SD Cook DG Strachan DP. Are noise and air pollution related to the incidence of dementia? A cohort study in London England. BMJ Open. (2018) 8:e022404. 10.1136/bmjopen-2018-022404 30206085 PMC6144407

[12] Chen H Kwong JC Copes R Hystad P van Donkelaar A Tu K. Exposure to ambient air pollution and the incidence of dementia: a population-based cohort study. Environ Int. (2017) 108:271–7. 10.1016/j.envint.2017.08.020 28917207

[13] Grande G Ljungman PLS Eneroth K Bellander T Rizzuto D. Association between cardiovascular disease and long-term exposure to air pollution with the risk of dementia. JAMA Neurol. (2020) 77:801–9. 10.1001/jamaneurol.2019.4914 32227140 PMC7105952

[14] Ilango SD Chen H Hystad P van Donkelaar A Kwong JC Tu K. The role of cardiovascular disease in the relationship between air pollution and incident dementia: a population-based cohort study. Int J Epidemiol. (2020) 49:36–44. 10.1093/ije/dyz154 31347651 PMC7124495

[15] Cancer IAfRo. Air Pollution and Cancer France. World Health Organization. (2013). Report No.: 978-92-832-2166-1.

[16] Oudin A Forsberg B Adolfsson AN Lind N Modig L Nordin M. traffic-related air pollution and dementia incidence in northern sweden: a longitudinal study. Environ Health Perspect. (2016) 124:306–12. 10.1289/ehp.1408322 26305859 PMC4786976

[17] Huat TJ Camats-Perna J Newcombe EA Valmas N Kitazawa M Medeiros R. Metal toxicity links to alzheimer's disease and neuroinflammation. J Mol Biol. (2019) 431:1843–68. 10.1016/j.jmb.2019.01.018 30664867 PMC6475603

[18] Brauer M Freedman G Frostad J van Donkelaar A Martin RV Dentener F. Ambient air pollution exposure estimation for the global burden of disease 2013. Environ Sci Technol. (2016) 50:79–88. 10.1021/acs.est.5b03709 26595236

[19] Fajersztajn L Saldiva P Pereira LAA Leite VF Buehler AM. Short-term effects of fine particulate matter pollution on daily health events in Latin America: a systematic review and meta-analysis. Int J Public Health. (2017) 62:729–38. 10.1007/s00038-017-0960-y 28255648

[20] Fajersztajn L Veras M Barrozo LV Saldiva P. Air pollution: a potentially modifiable risk factor for lung cancer. Nat Rev Cancer. (2013) 13:674–8. 10.1038/nrc3572 23924644

[21] Martins MCH Fatigati FL Ve'spoli TC Martins LC Pereira LAA Martins MA. Influence of socioeconomic conditions on air pollution adverse health effects in elderly people: an analysis of six regions in Sa~o Paulo, Brazil. J Epidemiol Community Health. (2004) 58:41–6. 10.1136/jech.58.1.41 14684725 PMC1757032

[22] Hajat A Diez-Roux AV Adar SD Auchincloss AH Lovasi GS O'Neill MS. Air pollution and individual and neighborhood socioeconomic status: evidence from the Multi-Ethnic Study of Atherosclerosis (MESA). Environ Health Perspect. (2013) 121:1325–33. 10.1289/ehp.1206337 24076625 PMC3855503

[23] Hajat A Hsia C O'Neill MS. Socioeconomic disparities and air pollution exposure: a global review. Curr Environ Health Rep. (2015) 2:440–50. 10.1007/s40572-015-0069-5 26381684 PMC4626327

[24] Laden F Schwartz J Speizer FE Dockery DW. Reduction in fine particulate air pollution and mortality: extended follow-up of the harvard six cities study. Am J Respir Crit Care Med. (2006) 173:667–72. 10.1164/rccm.200503-443OC 16424447 PMC2662950

[25] Clancy L Goodman P Sinclair H Dockery DW. Effect of air-pollution control on death rates in Dublin, Ireland: an intervention study. Lancet. (2002) 360:1210–4. 10.1016/S0140-6736(02)11281-5 12401247

[26] Rich DQ Kipen HM Huang W Wang G Wang Y Zhu P. Association between changes in air pollution levels during the Beijing Olympics and biomarkers of inflammation and thrombosis in healthy young adults. JAMA. (2012) 307:2068–78. 10.1001/jama.2012.3488 22665106 PMC4049319

[27] Yinon L Thurston G. An evaluation of the health benefits achieved at the time of an air quality intervention in three Israeli cities. Environ Int. (2017) 102:66–73. 10.1016/j.envint.2016.12.025 28237065 PMC5771478

[28] Gómez Peláez LM Santos JM de Almeida Albuquerque TT Reis NC Andreão WL de Fátima Andrade M. Air quality status and trends over large cities in South America. Environ Sci. Policy. (2020) 114:422–35. 10.1016/j.envsci.2020.09.009

[29] Gouveia N Kephart JL Dronova I McClure L Granados JT Betancourt RM. Ambient fine particulate matter in Latin American cities: levels, population exposure, and associated urban factors. Sci Total Environ. (2021) 772:145035. 10.1016/j.scitotenv.2021.145035 33581538 PMC8024944

[30] Jacobson Lda S Hacon Sde S de Castro HA Ignotti E Artaxo P Saldiva PH. Acute effects of particulate matter and black carbon from seasonal fires on peak expiratory flow of schoolchildren in the Brazilian Amazon. PLoS ONE. (2014) 9:e104177. 10.1371/journal.pone.0104177 25118606 PMC4131919

[31] Marlier ME Bonilla EX Mickley LJ. How do Brazilian fires affect air pollution and public health? Geohealth. (2020) 4:e2020GH000331. 10.1029/2020GH000331 33313462 PMC7698020

[32] Matsuda M Braga ALF Marquezini MV Monteiro MLR Saldiva PHN de Santos U. Occupational effect of sugarcane biomass burning on the conjunctival mucin profile of harvest workers and residents of an adjacent town-A Brazilian panel study. Exp Eye Res. (2020) 190:107889. 10.1016/j.exer.2019.107889 31801686

[33] Cancado JE Saldiva PH Pereira LA Lara LB Artaxo P Martinelli LA. The impact of sugar cane-burning emissions on the respiratory system of children and the elderly. Environ Health Perspect. (2006) 114:725–9. 10.1289/ehp.8485 16675427 PMC1459926

[34] Maas A Kothe H Centeno IP Leiva MJG Dalhoff1 K. Prevalence of chronic bronchitis and respiratory health profile of a population exposed to wood smoke in Nicaragua. J Health Pollut. (2020) 10:1–9. 10.5696/2156-9614-10.26.200607 32509408 PMC7269325

[35] Livingston G Sommerlad A Orgeta V Costafreda SG Huntley J Ames D. Dementia prevention, intervention, and care. Lancet. (2017) 390:2673–734. 10.1016/S0140-6736(17)31363-6 28735855

[36] Barnes DE Yaffe K. The projected effect of risk factor reduction on Alzheimer's disease prevalence. Lancet Neurol. (2011) 10:819–28. 10.1016/S1474-4422(11)70072-2 21775213 PMC3647614

[37] International AsD. From plan to impact Progress towards targets of the Global action plan on dementia (2018). London: Alzheimer's Disease International (ADI) (2018).

[38] Maher BA Ahmed IA Karloukovski V MacLaren DA Foulds PG Allsop D. Magnetite pollution nanoparticles in the human brain. Proc Natl Acad Sci USA. (2016) 113:10797–801. 10.1073/pnas.1605941113 27601646 PMC5047173

[39] Block ML Elder A Auten RL Bilbo SD Chen H Chen JC. The outdoor air pollution and brain health workshop. Neurotoxicology. (2012) 33:972–84. 10.1016/j.neuro.2012.08.014 22981845 PMC3726250

[40] Cacciottolo M Morgan TE Saffari AA Shirmohammadi F Forman HJ Sioutas C. Traffic-related air pollutants (TRAP-PM) promote neuronal amyloidogenesis through oxidative damage to lipid rafts. Free Radic Biol Med. (2020) 147:242–51. 10.1016/j.freeradbiomed.2019.12.023 31883973 PMC7075030

[41] Calderon-Garciduenas L Kavanaugh M Block M D'Angiulli A Delgado-Chavez R Torres-Jardon R. Neuroinflammation, hyperphosphorylated tau, diffuse amyloid plaques, and down-regulation of the cellular prion protein in air pollution exposed children and young adults. J Alzheimers Dis. (2012) 28:93–107. 10.3233/JAD-2011-110722 21955814

[42] Calderon-Garciduenas L Reynoso-Robles R Perez-Guille B Mukherjee PS Gonzalez-Maciel A. Combustion-derived nanoparticles, the neuroenteric system, cervical vagus, hyperphosphorylated alpha synuclein and tau in young Mexico City residents. Environ Res. (2017) 159:186–201. 10.1016/j.envres.2017.08.008 28803148

[43] Oudin A Segersson D Adolfsson R Forsberg B. Association between air pollution from residential wood burning and dementia incidence in a longitudinal study in Northern Sweden. PLoS ONE. (2018) 13:e0198283. 10.1371/journal.pone.0198283 29897947 PMC5999109

[44] Chen H Kwong JC Copes R Tu K Villeneuve PJ van Donkelaar A. Living near major roads and the incidence of dementia. Parkinson's disease, and multiple sclerosis: a population-based cohort study. Lancet. (2017) 389:718–26. 10.1016/S0140-6736(16)32399-6 28063597

[45] Kulick ER Elkind MSV Boehme AK Joyce NR Schupf N Kaufman JD. Long-term exposure to ambient air pollution, APOE-epsilon4 status, and cognitive decline in a cohort of older adults in northern Manhattan. Environ Int. (2020) 136:105440. 10.1016/j.envint.2019.105440 31926436 PMC7024003

[46] Kulick ER Wellenius GA Boehme AK Joyce NR Schupf N Kaufman JD. Long-term exposure to air pollution and trajectories of cognitive decline among older adults. Neurology. (2020) 94:e1782–e92. 10.1212/WNL.0000000000009314 32269113 PMC7274848

[47] Weuve J. Are we ready to call exposure to air pollution a risk factor for dementia? Neurology. (2020) 94:727–8. 10.1212/WNL.0000000000009318 32269115

[48] Livingston G Huntley J Sommerlad A Ames D Ballard C Banerjee S. Dementia prevention, intervention, and care: 2020 report of the Lancet Commission. Lancet. (2020) 396:413–46. 10.1016/S0140-6736(20)30367-6 32738937 PMC7392084

[49] FAO OPS WFP y UNICEF. Panorama de la seguridad alimentaria y nutrición en América Latina y el Caribe 2019. Santiago. 136 (2019). Licencia: CC BY-NC-SA 3.0 IGO.

[50] Aschner P. Obesity in Latin America BT-Metabolic Syndrome: A Comprehensive Textbook. Ahima RS, editor. Cham: Springer International Publishing. (2014) p. 1–8.

[51] Ng M Fleming T Robinson M Thomson B Graetz N Margono C. Global, regional, and national prevalence of overweight and obesity in children and adults during 1980-2013: a systematic analysis for the Global Burden of Disease Study 2013. Lancet. (2014) 384:766–81. 10.1016/S0140-6736(14)60460-8 24880830 PMC4624264

[52] Rao X Patel P Puett R Rajagopalan S. Air pollution as a risk factor for type 2 diabetes. Toxicol Sci. (2015) 143:231–41. 10.1093/toxsci/kfu250 25628401 PMC4306726

[53] Mullins J Bharadwaj P. Effects of Short-Term Measures to Curb Air Pollution: Evidence from Santiago, Chile. Am J Agric Econ. (2014) 97:1107–34.

[54] Blüher M. Obesity. global epidemiology and pathogenesis. Nat Rev Endocrinol. (2019) 15:288–98. 10.1038/s41574-019-0176-8 30814686

[55] Xue J Ideraabdullah FY. An assessment of molecular pathways of obesity susceptible to nutrient, toxicant and genetically induced epigenetic perturbation. J Nutr Biochem. (2016) 30:1–13. 10.1016/j.jnutbio.2015.09.002 27012616 PMC4808242

[56] An R Ji M Yan H Guan C. Impact of ambient air pollution on obesity: A systematic review. Int J Obes. (2018) 42:1112–26. 10.1038/s41366-018-0089-y 29795462

[57] Croft DP Zhang W Lin S Thurston SW Hopke PK Masiol M. The association between respiratory infection and air pollution in the setting of air quality policy and economic change. (2019). Ann Am Thorac Soc. 16:321–30. 10.1513/AnnalsATS.201810-691OC 30398895 PMC6394122

[58] Sanidas E Papadopoulos DP Grassos H Velliou M Tsioufis K Barbetseas J. Air pollution and arterial hypertension. A new risk factor is in the air. J Am Soc Hypertens. (2017) 11:709–15. 10.1016/j.jash.2017.09.008 28989071

[59] Jalaludin B Cowie C. Particulate air pollution and cardiovascular disease - It is time to take it seriously. Rev Environ Health. (2014) 29:129–32. 10.1515/reveh-2014-0031 24552964

[60] Kim H Kim WH Kim YY Park HY. Air Pollution and Central Nervous System Disease: A Review of the Impact of Fine Particulate Matter on Neurological Disorders. Front Public Health. (2020) 8:575330. 10.3389/fpubh.2020.575330 33392129 PMC7772244

[61] Béjot Y Reis J Giroud M Feigin V. A review of epidemiological research on stroke and dementia and exposure to air pollution. Int J Stroke. (2018) 13:687–95. 10.1177/1747493018772800 29699457

[62] Calderon-Garciduenas L Mora-Tiscareno A Ontiveros E Gomez-Garza G Barragan-Mejia G Broadway J. Air pollution, cognitive deficits and brain abnormalities: a pilot study with children and dogs. Brain Cogn. (2008) 68:117–27. 10.1016/j.bandc.2008.04.008 18550243

[63] Kim Y Myung W Won H-H Shim S Jeon HJ Choi J. Association between air pollution and suicide in South Korea: a nationwide study. PLoS ONE. (2015) 18 10:e0117929. 10.1371/journal.pone.0117929 25693115 PMC4333123

[64] Jianping L Changping Z Hongbing XD BR Shengcong L Tieci Y. Ambient air pollution is associated with HDL (high-density lipoprotein) dysfunction in healthy adult*s.* Arterioscler Thromb Vasc Biol. (2019) 39:513–22. 10.1161/ATVBAHA.118.311749 30700134

[65] Folino F Buja G Zanotto G Marras E Allocca G Vaccari D. Association between air pollution and ventricular arrhythmias in high-risk patients (ARIA study): a multicentre longitudinal study. Lancet Planet Heal. (2017) 1:e58–64. 10.1016/S2542-5196(17)30020-7 29851582

[66] Mishra S. Is smog innocuous? Air pollution and cardiovascular disease. Indian Heart J. (2017) 69:425–9. 10.1016/j.ihj.2017.07.016 28822504 PMC5560907

[67] Tonne C Melly S Mittleman M Coull B Goldberg R Schwartz J. A case-control analysis of exposure to traffic and acute myocardial infarction. Environ Health Perspect. (2007) 115:53–7. 10.1289/ehp.9587 17366819 PMC1797833

[68] Cai Y Zhang B Ke W Feng B Lin H Xiao J. Associations of short-term and long-term exposure to ambient air pollutants with hypertension: a systematic review and meta-analysis. Hypertension. (2016) 68:62–70. 10.1161/HYPERTENSIONAHA.116.07218 27245182

[69] Lim SS Vos T Flaxman AD Danaei G Shibuya K Adair-Rohani H. A comparative risk assessment of burden of disease and injury attributable to 67 risk factors and risk factor clusters in 21 regions, 1990-2010: a systematic analysis for the Global Burden of Disease Study 2010. Lancet. (2012) 380:2224–60. 10.1016/S0140-6736(12)61766-8 23245609 PMC4156511

[70] Brook RD. Cardiovascular effects of air pollution. Clin Sci (Lond). (2008) 115:175–87. 10.1042/CS20070444 18691154

[71] Mills NL Donaldson K Hadoke PW Boon NA MacNee W Cassee FR. Adverse cardiovascular effects of air pollution. Nat Clin Pract Cardiovasc Med. (2009) 6:36–44. 10.1038/ncpcardio1399 19029991

[72] Wellenius GA Schwartz J Mittleman MA. Air pollution and hospital admissions for ischemic and hemorrhagic stroke among medicare beneficiaries. STROKE. (2005) 36:2549–53. 10.1161/01.STR.0000189687.78760.47 16254223

[73] Yang BY Guo Y Bloom MS Xiao X Qian Z (Min) Liu E. Ambient PM1 air pollution, blood pressure, and hypertension: Insights from the 33 Communities Chinese Health Study. Environ Res. (2019) 170:252–9. Available from:. 10.1016/j.envres.2018.12.047 30597289

[74] Li N Chen G Liu F Mao S Liu Y Liu S. Associations between long-term exposure to air pollution and blood pressure and effect modifications by behavioral factors. Environ Res. (2020) 182:1–8. 10.1016/j.envres.2019.109109 32069739 PMC7043011

[75] Sun Q Hong X Wold LE. Cardiovascular effects of ambient particulate air pollution exposure. Circulation. (2010) 121:2755–65. 10.1161/CIRCULATIONAHA.109.893461 20585020 PMC2924678

[76] Iadecola C Yaffe K Biller J Bratzke LC Faraci FM Gorelick PB. Impact of hypertension on cognitive function: a scientific statement from the American heart association. Hypertension. (2016) 68: e67–e94. 10.1161/HYP.0000000000000053 27977393 PMC5361411

[77] Calderón-Garcidueñas L Reed W Maronpot RR Henríquez-Roldán C Delgado-Chavez R Calderón-Garcidueñas A. Brain inflammation and Alzheimer's-like pathology in individuals exposed to severe air pollution. Toxicol Pathol. (2004) 32:650–8. 10.1080/01926230490520232 15513908

[78] Calderón-Garcidueñas L Leray E Heydarpour P Torres-Jardón R Reis J. Air pollution, a rising environmental risk factor for cognition, neuroinflammation and neurodegeneration: The clinical impact on children and beyond. Rev Neurol. (2016) 172:69–80. 10.1016/j.neurol.2015.10.008 26718591

[79] Calderón-Garcidueñas L Torres-Jardón R Kulesza RJ Park S-B D'Angiulli A. Air pollution and detrimental effects on children's brain. The need for a multidisciplinary approach to the issue complexity and challenges. Front Hum Neurosci. (2014) 8:613. 10.3389/fnhum.2014.00613 25161617 PMC4129915

[80] Calderon-Garciduenas L Reynoso-Robles R Vargas-Martinez J Gomez-Maqueo-Chew A Perez-Guille B Mukherjee PS. Prefrontal white matter pathology in air pollution exposed Mexico City young urbanites and their potential impact on neurovascular unit dysfunction and the development of Alzheimer's disease. Environ Res. (2016) 146:404–17. 10.1016/j.envres.2015.12.031 26829765

[81] Hullmann M Albrecht C van Berlo D Gerlofs-Nijland ME Wahle T Boots AW. Diesel engine exhaust accelerates plaque formation in a mouse model of Alzheimer's disease. Part Fibre Toxicol. (2017) 14:35. 10.1186/s12989-017-0213-5 28854940 PMC5577845

[82] Morgan TE Davis DA Iwata N Tanner JA Snyder D Ning Z. Glutamatergic neurons in rodent models respond to nanoscale particulate urban air pollutants in vivo and in vitro. Environ Health Perspect. (2011) 119:1003–9. 10.1289/ehp.1002973 21724521 PMC3222976

[83] Dimakakou E Johnston HJ Streftaris G Cherrie JW. Exposure to environmental and occupational particulate air pollution as a potential contributor to neurodegeneration and diabetes: a systematic review of epidemiological research. Int J Environ Res Public Health. (2018) 15:1704. 10.3390/ijerph15081704 30096929 PMC6121251

[84] Ravi M Paul E. Stroke mortality associated with living near main roads in england and wales. Stroke. (2003) 34:2776–80. 10.1161/01.STR.0000101750.77547.11 14615623

[85] Lim Y-H Kim H Kim JH Bae S Park HY Hong Y-C. Air pollution and symptoms of depression in elderly adults. Environ Health Perspect. (2012) 120:1023–8. 10.1289/ehp.1104100 22514209 PMC3404652

[86] Anisman H Hayley S. Inflammatory factors contribute to depression and its comorbid conditions. Sci Signal. (2012) 5:pe45. 10.1126/scisignal.2003579 23033537

[87] Wei H Feng Y Liang F Cheng W Wu X Zhou R. Role of oxidative stress and DNA hydroxymethylation in the neurotoxicity of fine particulate matter. Toxicology. (2017) 380:94–103. 10.1016/j.tox.2017.01.017 28153600

[88] Dockery DW Pope CA. Acute respiratory effects of particulate air pollution. Annu Rev Public Health. (1994) 15:107–32. 10.1146/annurev.pu.15.050194.000543 8054077

[89] Juha P Annette P Gerard H Pekka T Bert B Jeroen de H. Particulate air pollution and risk of ST-segment depression during repeated submaximal exercise tests among subjects with coronary heart disease. Circulation. (2002) 106:933–8. 10.1161/01.CIR.0000027561.41736.3C 12186796

[90] Graber M Mohr S Baptiste L Duloquin G Blanc-Labarre C Mariet AS. Air pollution and stroke. A new modifiable risk factor is in the air. Rev Neurol. (2019) 175:619–24. 10.1016/j.neurol.2019.03.003 31153597

[91] Sahathevan R Brodtmann A Donnan GA. Dementia, stroke, and vascular risk factors a review. Int J Stroke. (2012) 7:61–73. 10.1111/j.1747-4949.2011.00731.x 22188853

[92] Shalev D Arbuckle MR. Metabolism and memory: obesity, diabetes, and dementia. Biol Psychiatry. (2017) 82:e81–3. 10.1016/j.biopsych.2017.09.025 29110819 PMC5712841

[93] Baron AD Steinberg HO Chaker H Leaming R Johnson A Brechtel G. Insulin-mediated skeletal muscle vasodilation contributes to both insulin sensitivity and responsiveness in lean humans. J Clin Invest. (1995) 96:786–92. 10.1172/JCI118124 7635973 PMC185264

[94] Gasparotto J Chaves PR da Boit Martinello K Silva Oliveira LF Gelain DP Fonseca Moreira JC. Obesity associated with coal ash inhalation triggers systemic inflammation and oxidative damage in the hippocampus of rats. Food Chem Toxicol. (2019) 133:110766. 10.1016/j.fct.2019.110766 31430511

[95] D'Mello C Le T Swain MG. Cerebral microglia recruit monocytes into the brain in response to tumor necrosis factoralpha signaling during peripheral organ inflammation. J Neurosci. (2009) 18 29:2089–102. 10.1523/JNEUROSCI.3567-08.2009 19228962 PMC6666330

[96] Calderon-Garcidueñas L Osorno-Velazquez A Bravo-Alvarez H Delgado-Chavez R Barrios-Marquez R. Histopathologic changes of the nasal mucosa in southwest Metropolitan Mexico City inhabitants. Am J Pathol. (1992) 140:225–32. 1731527 PMC1886244

[97] Sleeman KE de Brito M Etkind S Nkhoma K Guo P Higginson IJ. The escalating global burden of serious health-related suffering: projections to 2060 by world regions. age groups, and health conditions. Lancet GlobHealth. (2019) 7: e883–92. 10.1016/S2214-109X(19)30172-X 31129125 PMC6560023

[98] Richard B Chen H Szyszkowicz M Fann N Hubbell B Pope CA Apte JS. Global estimates of mortality associated with long-term exposure to outdoor fine particulate matter. Proc Natl Acad Sci USA. (2018) 115:9592–97. 10.1073/pnas.1803222115 30181279 PMC6156628

[99] Gładka A Rymaszewska J Zatoński T. Impact of air pollution on depression and suicide. Int J Occup Med Environ Health. (2018) 31:711–721. 10.13075/ijomeh.1896.01277 30281038

[100] Kilian J Kitazawa M. The emerging risk of exposure to air pollution on cognitive decline and Alzheimer's disease - Evidence from epidemiological and animal studies. Biomed J. (2018) 41:141–62. 10.1016/j.bj.2018.06.001 30080655 PMC6138768

[101] Shou Y Huang Y Zhu X Liu C Hu Y Wang H. A review of the possible associations between ambient PM2.5 exposures and the development of Alzheimer's disease. Ecotoxicol Environ Saf. (2019) 174:344–52. 10.1016/j.ecoenv.2019.02.086 30849654

[102] Calderon-Garciduenas L Gonzalez-Maciel A Reynoso-Robles R Kulesza RJ Mukherjee PS Torres-Jardon R. Alzheimer's disease and alpha-synuclein pathology in the olfactory bulbs of infants, children, teens and adults < /=40 years in Metropolitan Mexico City. APOE4 carriers at higher risk of suicide accelerate their olfactory bulb pathology. Environ Res. (2018) 166:348–62. 10.1016/j.envres.2018.06.027 29935448

[103] Villa N Leticia C Zilli V. Levels of Polonium-210 in brain and pulmonary tissues : Preliminary study in autopsies conducted in the city of Saõ Paulo, Brazil. Sci. Rep. (2020). 10:180. 10.1289/isee.2020.virtual.P-0612 31932745 PMC6957520

[104] Calderón-Garcidueñas L Mora-Tiscareño A Franco-Lira M Zhu H Lu Z Solorio E. Decreases in short term memory, IQ, and altered brain metabolic ratios in urban Apolipoprotein ε4 children exposed to air pollution. J Alzheimers Dis. (2015) 45:757–70. 10.3233/JAD-142685 25633678

[105] Calderon-Garciduenas L Serrano-Sierra A Torres-Jardon R Zhu H Yuan Y Smith D. The impact of environmental metals in young urbanites' brains. Exp Toxicol Pathol. (2013) 65:503–11. 10.1016/j.etp.2012.02.006 22436577 PMC3383886

[106] Burns J Boogaard H Polus S Pfadenhauer LM Rohwer AC van Erp AM. Interventions to reduce ambient air pollution and their effects on health: An abridged Cochrane systematic review. Environ Int. (2020) 135:105400. 10.1016/j.envint.2019.105400 31855800

[107] Riojas-Rodríguez H SdSA Texcalac-Sangrador JL Moreno-Banda GL. Air pollution management and control in Latin America and the Caribbean: implications for climate change. Rev Panam Salud Publica. (2016) 40:150–9. 10.1289/isee.2016.4675 27991972

[108] Green J SS. Air Quality in Latin America: An Overview. Clean Air Institute. (2013).

[109] World Health Organization. Regional Office for Europe. (2006). Air quality guidelines: global update 2005: particulate matter, ozone, nitrogen dioxide and sulfur dioxide. World Health Organization. Regional Office for Europe. Available online at: https://apps.who.int/iris/handle/10665/107823

[110] Mullins J Bharadwaj P. Effects of Short-Term Measures to Curb Air Pollution: Evidence from Santiago, Chile. Am J Agric Econ. (2014) 97:1107–34. 10.1093/ajae/aau081

[111] Gramsch E Le Nir G Araya M Rubio MA Moreno F Oyola P. Influence of large changes in public transportation (Transantiago) on the black carbon pollution near streets. Atmos Environ. (2013) 65:153–63. 10.1016/j.atmosenv.2012.10.006

[112] Gallego F Montero J-P Salas C. The effect of transport policies on car use: evidence from Latin American cities. J Public Econ. (2013) 107:47–62. 10.1016/j.jpubeco.2013.08.007

[113] Andrade MdF Kumar P de Freitas ED Ynoue RY Martins J Martins LD. Air quality in the megacity of São Paulo: evolution over the last 30 years and future perspectives. Atmos Environ. (2017) 159:66–82. 10.1016/j.atmosenv.2017.03.051

[114] Sisa I Abeyá-Gilardon E Fisberg RM Jackson MD Mangialavori GL Sichieri R. Impact of diet on CVD and diabetes mortality in Latin America and the Caribbean: a comparative risk assessment analysis. Public Health Nutr. (2020) 2016:2577–91. 10.1017/S1368980020000646 32489172 PMC7710925

[115] GDB Results Tool 2021. University of Washington Institute of Health Metrics and Evaluation. (2021).

[116] Barceló A Aedo C Rajpathak S Robles S. The cost of diabetes in Latin America and the Caribbean. Bull World Health Organ. (2003) 81:19–27. 12640472 PMC2572319

[117] Diabetes TL. Obesity prevention in Latin America: Now is the time. Lancet Diabetes Endocrinol. (2014) 2:263. 10.1016/S2213-8587(14)70079-8 24703034

[118] Mukadam N Sommerlad A Huntley J Livingston G. Population attributable fractions for risk factors for dementia in low-income and middle-income countries: an analysis using cross-sectional survey data. Lancet Glob Health. (2019) 7:e596–e603. 10.1016/S2214-109X(19)30074-9 31000129 PMC7617123

